# Prevalence and factors of behavioral intention of gaming time reduction among high-risk adolescents in China: an application of the Conservation of Resource Theory

**DOI:** 10.1186/s12888-025-07769-1

**Published:** 2026-01-30

**Authors:** Yanqiu Yu, Joseph T. F. Lau

**Affiliations:** 1 School of Public Health, Fudan University, Shanghai, China; 2 School of Mental Health, Wenzhou Medical University, Wenzhou, China; 3 Zhejiang Provincial Clinical Research Center for Mental Disorders, The Affiliated Wenzhou Kangning Hospital, Wenzhou Medical University, Wenzhou, China; 4 Centre for Health Behaviours Research, Jockey Club School of Public Health and Primary Care, The Chinese University of Hong Kong, Hong Kong, China

**Keywords:** Behavioral intention, Gaming time reduction, Conservation of resource theory, Adolescent, Internet gaming disorder

## Abstract

**Background:**

Gaming time reduction has been effective in reducing internet gaming disorder (IGD). This study investigated the prevalence of behavioral intention of gaming time reduction (BI-GTR) among middle school students who self-perceived having IGD. Based on the Conservation of Resource (COR) theory, the mediations between personal/interpersonal resource losses due to gaming time reduction and BI-GTR via perceived stress due to gaming time reduction were investigated.

**Methods:**

A cross-sectional, anonymous survey was conducted among Chinese adolescent internet gamers with self-perception of IGD in Chengdu, China, from December 2019 to January 2020 (*n* = 485). Self-perception of IGD was assessed by a screening question “Do you perceive yourself having IGD? (yes/no response options)”, while probable IGD was assessed by the 9-item DSM-5 Checklist.

**Results:**

Among all participants with self-perception of IGD, the prevalence of BI-GTR was 67.4%; it was significantly lower in the probable IGD group than the non-probable IGD group (56.2% versus 73.4%, OR = 1.63, 95% CI: 1.27, 2.08). Multivariate logistic regression analyses showed that, with the adjustment of background factors, personal resource losses (ORa = 0.93, 95% CI: 0.91, 0.95), interpersonal resource losses (ORa = 0.92, 95% CI: 0.90, 0.95), and perceived stress (ORa = 0.60, 95% CI: 0.49, 0.72) were significantly and negatively associated with BI-GTR. Path analysis, adjusting for background factors as well, revealed that the negative association between personal resource losses and BI-GTR was significantly and partially mediated via perceived stress (*β* = − 0.07; *p* = 0.023; mediation effect size = 15.6%); The similar indirect effects of perceived stress between interpersonal resource losses and BI-RGT were, however, statistically non-significant (*β* = − 0.01; *p* = 0.874). Multigroup path analysis further showed that the mediation mechanisms were invariant between the probable IGD and non-probable IGD groups.

**Conclusions:**

The novel findings about the prevalence of BI-GTR, its associated factors, and mediations among Chinese adolescents with self-perception of IGD have implications for future research/interventions. It has extended the application of COR theory to studying health-seeking behaviors. Future confirmation and interventional studies are required.

**Clinical trial number:**

Not applicable.

## Introduction

Adolescents are vulnerable to internet gaming disorder (IGD) [1] and its negative consequences in terms of physical health, mental health, and social relationships [2–4]. IGD has become a global public health issue and was included in *the International Classification of Diseases*,* 11th Revision (ICD-11)* [5]. Although long internet gaming time is not a diagnostic criterion of IGD, it was strongly associated with IGD [6] and various negative mental/physical health outcomes [7]. Extant interventions (e.g., cognitive-behavioral therapy) aimed at reducing IGD symptoms through the reduction of internet gaming time [8]. This approach demonstrated effectiveness in reducing IGD symptoms [9, 10]. It is hence important to understand the key determinants and related mechanisms underlying gaming time reduction. Such studies are lacking.

Behavioral intention is a predictor of actual behavior, both theoretically and empirically [11, 12]. According to the Theory of Planned Behavior, behavioural intention is a determinant of the behaviour of concern [11] and a commonly used dependent variable in research on addictive behaviors, including internet addiction [12–14]. Notably, in cross-sectional studies, it is hard to discern the direction of a significant association between a risk factor and a behavior (e.g., the potential bidirectionality between perceptions and health behaviors). As behavioral intention refers to the chance of performing a behavior in the future, it is not a temporal precedent of the independent variables (e.g., perceptions). The interpretation of an association between an independent variable and a behavioral intention is hence less subject to temporal bias. In this study, behavioral intention of gaming time reduction (BI-GTR) was used as the dependent variable.

The well-established Conservation of Resources (COR) Theory served as the theoretical framework of this study. The COR theory postulates that people strive to retain, protect, and build various resources (e.g., personal and interpersonal resources); when they experience actual or perceived resource losses, stress ensues and can lead to adverse health outcomes [15–18]. Internet gaming could provide important personal and interpersonal resources, as literature has documented that adolescents attain various rewards from internet gaming, such as self-esteem and peer recognition [19]. Gaming time reduction can thus be a potential stressor, as it may increase perceived losses of the gaming-related personal and interpersonal resources [19]. According to the COR theory [15–18], perceived resource losses would enhance perceived stress, which would in turn increase the risk of adopting maladaptive coping responses and mental distress. To avoid resource losses and resultant stress, those anticipating resource losses due to gaming time reduction might be less likely to indicate BI-GTR. A mediation mechanism is hence hypothesized that perceived personal/interpersonal resource losses due to gaming time reduction (PRL-GTR/IRL-GTR) may increase perceived stress due to gaming time reduction (PS-GTR; a mediator), which would in turn reduce BI-GTR. The strengths of such a mediation effect may, however, differ between those with or without IGD, as the former group might perceive stronger resource losses than the latter and find it more stressful. Furthermore, with impaired control, IGD cases may find it more difficult to reduce their gaming time. This invariance hypothesis has not been tested in literature.

The present study investigated the level of BI-GTR among junior middle school students with self-perception of IGD (SP-IGD) in Chengdu, China. This subgroup was selected because awareness of one’s IGD status may be a prerequisite for gaming time reduction. As adolescents with SP-IGD did not necessarily know their actual IGD status, SP-IGD likely had greater influence on subsequent gaming time reduction than diagnosed IGD status. Moreover, individuals with SP-IGD tended to report longer gaming time and more severe IGD symptoms [20], indicating a correspondingly stronger need for managing gaming time. Associated factors of BI-GTR were also investigated, including PRL-GTR, IRL-GTR, and PS-GTR. The mediation hypothesis that the associations between PRL-GTR/IRL-GTR and BI-GTR would be mediated by PS-GTR was further examined. Specifically, it was hypothesized that PRL-GTR/IRL-GTR would increase PS-GTR, which would in turn decrease BI-GTR. Multigroup analysis was performed to inspect whether the proposed mediation model would be invariant by IGD status.

## Methods

### Participants and data collection

A cross-sectional survey was conducted among junior middle school students in Chengdu, China, from December 2019 to January 2020. Three schools were conveniently selected and participated in the present study. All Grade 7–9 students of these schools were invited to participate. The students were asked to self-administer an anonymous, structured questionnaire in a classroom setting and in the absence of teachers. Prior to the survey, students were briefed by well-trained field workers about the survey’s objectives, content, and logistics. It was emphasized that participation was voluntary and that refusal or quitting at any time would bear no adverse consequences. To maintain anonymity, written informed consent was not required. The fieldworkers explained to the students that the return of the completed questionnaire implied informed consent to join the study. The same message was printed on the cover page of the questionnaire. Instead of signing a written consent form, parents were informed about the study and that they could have their children opt out of the study by filling out a form without any adverse consequences. Similar methods have been used in other studies [21, 22]. No parental opt-out form was received. No incentives were given to the participants. The present study was conducted in accordance with the Declaration of Helsinki and was approved by the Survey and Behavioral Research Ethics Committee of the Chinese University of Hong Kong (No. SBRE-18-430; Date of approval: February 27, 2019).

**Fig. 1:**
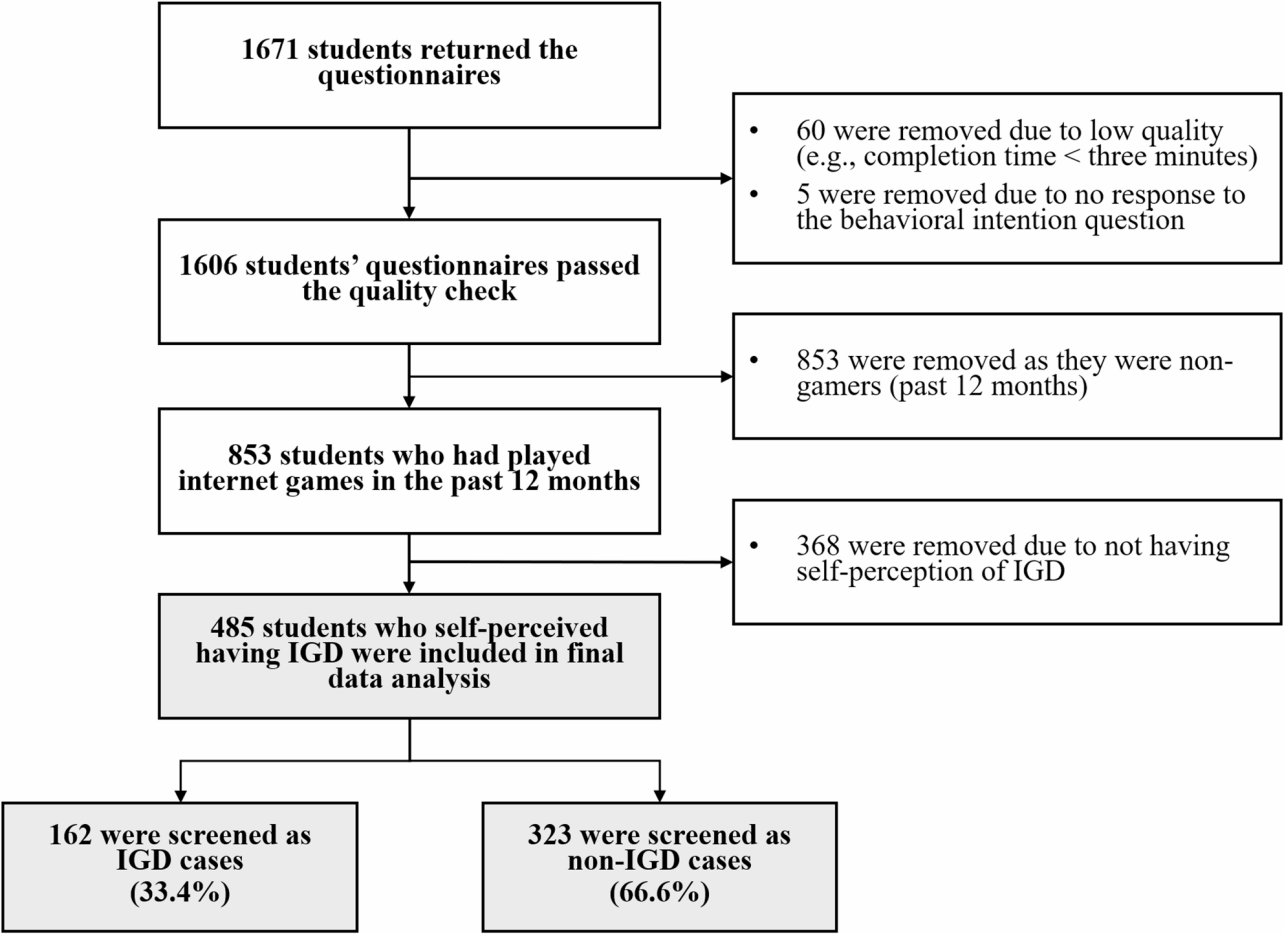
Flowchart of participant inclusion/exclusion process (IGD = Internet gaming disorder)

A total of 1,671 completed questionnaires were collected, 60 (3.6%) of which were excluded from data analysis due to quality control (i.e., missing values were found in at least 20% of all question items); five (0.3%) were removed due to no response to the key variable of BI-GTR; 853 (51.0%) were removed as they had not played internet games in the past 12 months. Of the remaining 853 internet gamers, a screening question (“Do you perceive yourself having IGD currently?”; yes/no response options) was used to identify those with SP-IGD; 485 (56.9%) indicated SP-IGD and comprised the final sample for statistical analysis. Figure 1 presents the flowchart of the above participant inclusion/exclusion process.

### Measurements

#### Socio-demographics

Information was collected on age, gender (female/male), whether living with both parents (yes/no), single-parent family (yes/no), and perceived household financial situation relative to classmates (good/very good/average/poor/very poor). These variables were treated as confounders, as they were significantly associated with perceived resource losses, perceived stress, and behavioral intention of self-corrective behaviors for internet addiction [12, 23, 24].

#### Behavioral intention of gaming time reduction (BI-GTR)

The item assessed whether the participant intended to reduce his/her internet gaming time in the next 12 months (yes/no response options).

#### Probable IGD

The 9-item DSM-5 IGD Checklist was used [25]. The IGD symptoms included preoccupation, withdrawal, tolerance, inability to control gaming behavior, prioritization of gaming over other activities, continuation of gaming despite adverse consequences, deception of gaming time, gaming as a means of avoidance, and significant negative consequences due to gaming (yes/no response options). The endorsement of five or more IGD symptoms in the past 12 months would indicate probable IGD (positive/negative). The Chinese version of the checklist has been validated among adolescents in China and showed satisfactory psychometric properties [26]. The Cronbach’s alpha of the checklist was 0.78 in this study.

#### Perceived personal/interpersonal resource loss due to gaming time reduction (PRL-GTR/IRL-GTR)

The Resource Loss due to the Reduction in Gaming Time Scale has been validated among Chinese adolescents and showed satisfactory psychometric properties [23]. Its two subscales, the 11-item PRL-GTR and the 8-item IRL-GTR subscales, were used in this study. Sample items included “reducing gaming time makes me lose a sense of achievement” for PRL-GTR and “Reducing gaming time makes me unable to meet new friends” for IRL-GTR. The items were rated on 5-point Likert scales (0 = no loss at all to 4 = loss to an extremely great extent). Higher scores indicated higher levels of PRL-GTR and IRL-GTR, respectively. In this study, the Cronbach’s alpha values of the two subscales were 0.96 and 0.94, respectively.

#### Perceived stress due to gaming time reduction (PS-GTR)

The item assessed the level of perceived stress in the case of reducing gaming time (0 = none to 4 = extremely high). A number of studies used a similar single item to assess perceived stress [27, 28].

### Statistical analysis

Pearson/Spearman correlation coefficients were derived to test the interrelationships among PRL-GTR, IRL-GTR, PS-GTR, and BI-GTR. Multivariate logistic regression was used to examine the significance and directions of the potential factors of BI-GTR, after adjusting for background factors (age, gender, whether living with both parents, single-parent family, and relative household financial situations). Path analysis was fit to test the underlying mechanisms between PRL-GTR/IRL-GTR and BI-GTR via PS-GTR, after adjusting for background factors (same as the ones in logistic regression). The Weighted Least Squares Mean and Variance method was used for estimation. Furthermore, multigroup path analysis was fit to examine the invariance of each structural/indirect path of the mediation mechanism between probable IGD and non-probable IGD groups. A series of models, each constraining a specific individual path (e.g., PRL-GTR → PS-GTR), were compared to the unconstrained model, testing all the paths freely. The significance level was set at *p-*value < 0.05 in the Wald test to denote a significant moderation effect of probable IGD on the tested path. SPSS 23.0 and Mplus 7.0 were used for statistical analysis. Statistical significance was determined by a two-tailed *p*-value < 0.05.

## Results

### Participants’ characteristics

Of all participants with SP-IGD, the mean (SD; range) age was 13.6 (0.9; 12–18) years. Over half were males (63.1%). More than one-fifth were not living with both parents (29.3%) and were from a single-parent family (21.2%). Close to one-fifth (15.3%) self-reported poor or very poor household financial situation relative to their classmates (See Table 1). The prevalence of probable IGD was 33.4%. The prevalence of BI-GTR was 67.4%; it was 56.2% in the probable IGD versus 73.4% in the non-probable IGD group (OR = 1.63, 95% CI: 1.27, 2.08). The mean (SD; range) scores of PRL-GTR, IRL-GTR, and PS-GTR were 12.1 (10.5; 0–44), 8.2 (7.8; 0–32), and 1.4 (1.1; 0–4), respectively; these results were not tabulated.

**Table 1:** Participants’ characteristics (*n* = 485)

	*n*	%
**Socio-demographics**		
Gender		
Female	172	35.5
Male	306	63.1
Missing data	7	1.4
Living with both parents		
Yes	336	69.3
No	142	29.3
Missing data	7	1.4
Single-parent family		
No	376	77.5
Yes	103	21.2
Missing data	6	1.2
Household financial situation relative to classmates		
Good / Very good	108	22.3
Average	298	61.4
Poor / Very poor	74	15.3
Missing data	5	1.0
**Probable IGD** ^**¶**^		
Negative	323	66.6
Positive	162	33.4
**BI-GTR**		
No	158	32.6
Yes	327	67.4

Note. IGD = Internet gaming disorder; BI-GTR = Behavioral intention of gaming time reduction. ^¶^, Probable IGD was defined as those endorsing five or more (out of nine) items of the DSM-5 IGD checklist

### Correlation analysis

As shown in Table 2, PRL-GTR, IRL-GTR, and PS-GTR were all negatively correlated with BI-GTR (Spearman *r* = − 0.31 to − 0.26). PRL-GTR and IRL-GTR were positively correlated with each other (Pearson *r* = 0.87), and both of them were positively correlated with PS-GTR (Pearson *r* = 0.41 and 0.41, respectively).

**Table 2:** Correlation analysis (*n* = 485)

	1	2	3
1. Perceived personal resource loss due to gaming time reduction	-		
2. Perceived interpersonal resource loss due to gaming time reduction	0.87***	-	
3. Perceived stress due to gaming time reduction	0.48***	0.41***	-
4. Behavioral intention of gaming time reduction	–0.31***	–0.26***	–0.26***

Note. ***, *p* < 0.001. Spearman correlation coefficients were generated between BI-GTR and the other three variables, while Pearson correlation coefficients were generated among the three psychosocial variables

### Adjusted associations

Multivariate logistic regression analyses, adjusting for the background factors, found that PRL-GTR (ORa = 0.93, 95% CI: 0.91, 0.95), IRL-GTR (ORa = 0.92, 95% CI: 0.90, 0.95), PS-GTR (ORa = 0.60, 95% CI: 0.49, 0.72), and probable IGD (ORa = 0.50, 95% CI: 0.33, 0.75) were negatively associated with BI-GTR (see Table 3).

**Table 3:** Multivariate logistic regression analysis (*n* = 485)

	Behavioral intention of gaming time reduction
	ORa (95% CI)
Perceived personal resource loss due to gaming time reduction	0.93 (0.91, 0.95)
Perceived interpersonal resource loss due to gaming time reduction	0.92 (0.90, 0.95)
Perceived stress due to gaming time reduction	0.60 (0.49, 0.72)
Internet gaming disorder	0.50 (0.33, 0.75)

Note. The models were adjusted for background factors, including age, gender, whether living with both parents, single-parent family, and relative household financial situation

### Path analysis

**Fig. 2:**
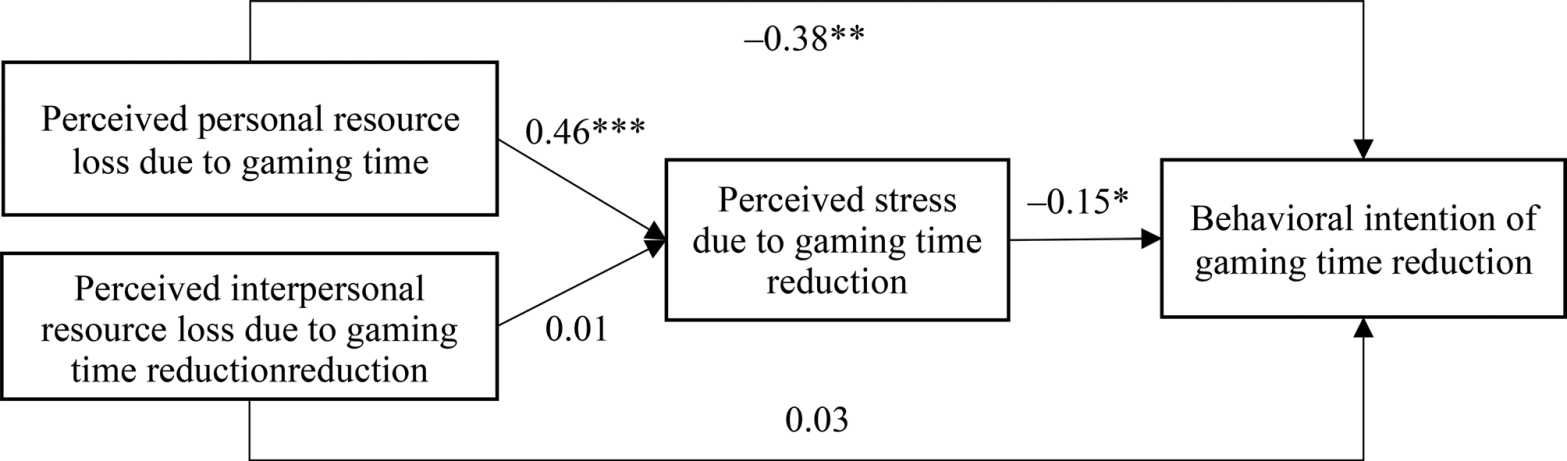
Path analysis (*, *p* < 0.05; **, *p* < 0.01; ***, *p* < 0.001; this model was adjusted for background factors, including age, gender, whether living with both parents, single-parent family, and relative household financial situation; standardized coefficients were reported)

Figure 2 presents path analysis results regarding the mediation effect of PS-GTR between PRL-GTR/IRL-GTR and BI-GTR, after adjusting for background factors of age, gender, whether living with both parents, a single-parent family, and relative household financial situation. First, both the direct effect (*β* = − 0.38; *p* < 0.001) and indirect effect via PS-GTR (*β* = − 0.07; *p* = 0.023) from PRL-GRT to BI-GTR were of statistical significance, denoting a partial mediation effect of PS-GTR (mediation effect size = 15.6%). In contrast, both the direct effect (*β* = 0.03; *p* = 0.762) and indirect effect via PS-GTR (*β* = − 0.01; *p* = 0.874) were statistically non-significant. Accordingly, PRL-GTR (*β* = 0.46; *p* < 0.001), but not IRL-GTR (*β* = 0.01; *p* = 0.847), was positively associated with PS-GTR, which in turn was negatively associated with BI-GTR (*β* = − 0.15; *p* = 0.014).

### Multigroup path analysis by probable IGD status

It is seen from Table 4 that the moderation effects of probable IGD for all the structural and indirect paths were statistically non-significant, indicating that the direction and strengths of the paths shown in Fig. 2 were statistically invariant between the probable IGD group and the non-IGD group.

**Table 4:** Multigroup path analysis by probable IGD status (*n* = 485)

	Probable IGD		Non-probable IGD		Wald test		
	β	*p*	β	*p*	Estimate	*p*	Invariance
**Structural path**							
PRL-GTR → PS-GTR	0.38	0.002	0.23	0.004	0.77	0.382	Supported
IRL-GTR → PS-GTR	0.17	< 0.001	0.16	< 0.001	0.01	0.913	Supported
PS-GTR → BI-GTR	–0.03	< 0.001	–0.03	< 0.001	0.45	0.502	Supported
PRL-GTR → BI-GTR	–0.23	0.191	–0.53	< 0.001	2.07	0.151	Supported
IRL-GTR → BI-GTR	–0.14	0.003	–0.19	< 0.001	1.18	0.277	Supported
**Indirect path**							
PRL-GTR → PS-GTR → BI-GTR	–0.01	0.015	–0.01	0.011	0.16	0.689	Supported
IRL-GTR → PS-GTR → BI-GTR	–0.01	0.005	–0.01	< 0.001	0.27	0.602	Supported

Note. IGD = Internet gaming disorder; PRL-GTR = Perceived personal resource loss due to gaming time reduction; IRL-GTR = Perceived interpersonal resource loss due to gaming time reduction; BI-GTR = Behavioral intention of gaming time reduction

## Discussion

This study was the first to observe a moderately high prevalence of BI-GTR among adolescent gamers with SP-IGD. Partially supporting the COR theory, path analysis found that PRL-GTR, but not IRL-GTR, was positively associated with PS-GTR, which was in turn negatively associated with BI-GTR, denoting a partial mediation effect of PS-GTR between PRL-GTR and BI-GTR. Multigroup path analysis further revealed that the structural/indirect paths in the proposed mediation mechanisms were invariant between those with and without probable IGD.

About one-third of the participants had probable IGD, which was much higher than that of 13% among general middle school students in previous studies [29]. The large difference is highly likely due to the sample characteristics of individuals with SP-IGD in this study, as SP-IGD status was positively associated with IGD [20, 30]. Notably, about two-thirds of those with SP-IGD did not have IGD (‘false positives’). Future studies are required to clarify whether the ‘false positives’ of SP-IGD were associated with equating intensive internet gaming with IGD erroneously [31] or incorrect judgments made by significant others (e.g., parents and peers).

In this study, the prevalence of BI-GTR was moderately high (67.4%). It suggests that many adolescents with SP-IGD might not find their internet gaming condition ideal and would like to rectify the situation. This observation was encouraging, as motivation is a key prerequisite for behavioural change [11]. Qualitative and quantitative research are required to understand the reasons behind BI-GTR and the obstacles to translating BI-GTR into actual behavior. Universal prevention may consider increasing the awareness of IGD. Interestingly, those with probable IGD exhibited lower BI-GTR than those without probable IGD. Possibly, IGD cases have addictive characteristics (e.g., tolerance and impaired control) that might hinder protective behaviors. Nonetheless, the prevalence of BI-GTR among those with both SP-IGD and probable IGD was still quite high (56.2%). Thus, many students simultaneously engaged in problematic gaming but intended to reduce gaming time. This reflects the cognitive dissonance status, a situation involving conflicting beliefs and behaviors, which would result in mental discomfort [32]. To reduce cognitive dissonance and resultant discomfort, individuals are expected to change either their beliefs (e.g., maladaptive cognitions justifying the problematic gaming patterns) or behaviors (e.g., protective behaviors of gaming time reduction) [32]. As the latter is difficult to achieve, adolescents might develop maladaptive cognitions instead, which potentially explain the commonly observed maladaptive cognitions among problematic gamers.

Negative associations were observed between PRL-GTR/IRL-GTR and BI-GTR, which corroborated previous results [23]. PRL-GRT and IRL-GRT can be seen as negative outcome expectancies of gaming time reduction. According to Social Cognitive Theory, negative outcome expectancies would reduce the likelihood of practicing the behavior [33]. It may be potentially useful to increase BI-GTR by reducing PRL-GTR/IRL-GTR. In addition, PRL-GTR and IRL-GTR are reflections of potential rewards from internet gaming. Extant theories (e.g., the Interaction of Person-Affect-Cognition-Execution model) [34] and empirical studies [22, 35] have reported that overreliance on internet gaming to obtain rewards was positively associated with IGD. Internet gaming might be used to fulfil adolescents’ unmet needs. Without addressing those needs, interventions on gaming time reduction may cause psychological reactance and even disrupt parent-child relationships [36]. Future interventions should consider adding elements to compensate for PRL-GTR and IRL-GTR, such as provision of alternative activities (e.g., art and exercise) and social skill training [8].

The positive association between PS-GTR and BI-GTR indicates that PS-GTR would undermine motivation to reduce gaming time. Thus, stress management may increase BI-GTR. The hypothesis that PS-GTR mediated the association between PRL-GTR was supported by the data. Although the COR theory does not postulate directly that perceived resource losses would lead to behavioral changes, previous studies have shown that mental distress may reduce health-seeking behaviours (gaming time reduction in this case) [37]. Thus, the significant mediation can be an extension of applying the COR theory to IGD research and behavioral changes. Attention is needed that the observed mediation effect size was moderate. Thus, other potential mediators need to be considered (e.g., mental distress and coping resources/styles).

The similar direct and mediation effects involving IRL-GTR were statistically non-significant. Different types of resource losses might induce differential impacts on mental health and health-related behaviors. The current findings suggest that concerns about losing personal resources seemed more influential on BI-GTR than those about losing interpersonal resources. Although internet-based peer support is important to adolescents, internet gaming might only be one of many sources of peer support. In contrast, personal resources obtained via internet gaming (e.g., sense of achievement and self-esteem) might be less replaceable. Notably, simple correlation analyses found significant correlations between IRL-GTR and PS-/BI-GTR, which were non-significant in path analysis. A methodological reminder is that, since PRL-GTR was associated with IRL-GTR, the effect of IRL-GTR might become smaller or non-significant after adjusting for PRL-GTR in path analysis.

The null hypothesis that the mediation model would be invariant by IGD status was supported by the data. PRL-GTR and IRL-GTR had similar effects on BI-GTR among those with and without IGD. Thus, interventions modifying perceived resource losses to increase BI-GTR among those with SP-IGD are warranted, disregarding their actual IGD status.

This study was subjected to several limitations. First, the self-administered questionnaire may incur reporting bias, such as recall bias and social desirability bias. Second, the cross-sectional study design renders the inability to make temporal or causal inferences. The findings need to be verified by future longitudinal and intervention studies. Third, the participating schools were conveniently selected from one Chinese city; generalization of the results to other regions or populations in and outside China should be made with caution. Fourth, the measurement of PS-GTR was constructed for this study. The measurement of probable IGD was based on DSM-5 instead of ICD-11; the prevalence of IGD may have been inflated [38]. Fifth, some domains of PRL-GTR (e.g., material losses and emotional losses) might not be included in this study. Sixth, some probable IGD cases without SP-IGD were excluded in this study, but the number should be relatively small. Last, some mediators between PPL-GTR/PIL-GTR and BI-GTR were not investigated in this study (e.g., mental distress and coping strategies).

In conclusion, this study reported high prevalence of BI-GTR among adolescent gamers with SP-IGD in China, and this prevalence was higher among those without probable IGD than those with probable IGD. An important finding is that PRL-GTR, but not IRL-GTR, was negatively associated with BI-GTR. The reasons behind the discrepancy need to be clarified in future studies. Interventions need to compensate for such potential resource losses by offering alternative rewarding activities. Although PS-GTR mediated between PRL-GTR and BI-GTR, the mediation effect size was not large. This study was thus only a starting point to understand the mechanisms explaining the development of BI-GTR, as well as the extension of applying the COR theory to health-seeking behaviors. Future longitudinal and intervention studies are needed to verify the current findings and explore other potential mediators between resource losses and behavioral outcomes.

## Data Availability

Data is available on reasonable request from the corresponding author.
